# Anticancer Activity of *Jania rubens* in HCT-116 Cells via EMT Suppression, TET Downregulation, and ROS-Mediated Cytotoxicity

**DOI:** 10.3390/biom15101361

**Published:** 2025-09-25

**Authors:** Zeina Radwan, Rayan Kassir, Fouad Al Feghaly, Rouaa Zaiter, Mira Abou Daher, Rabih Roufayel, Ziad Fajloun, Hiba Mawlawi, Marwan El-Sabban, Zeina Dassouki

**Affiliations:** 1 Department of Anatomy, Cell Biology, and Physiological Sciences, Faculty of Medicine, American University of Beirut, Beirut P.O. Box 55251 Sin El Fil, Lebanon; zar04@mail.aub.edu (Z.R.);; 2 Laboratory of Applied Biotechnology (LBA3B), AZM Center for Research in Biotechnology and Its Applications, Doctoral School for Sciences and Technology, Lebanese University, Tripoli 1300, Lebanon; 3 College of Engineering and Technology, American University of the Middle East, Egaila 54200, Ahmadi, Kuwait; 4 Faculty of Sciences 3, Lebanese University, Michel Slayman Tripoli Campus, Ras Maska, Tripoli 1300, Lebanon; 5 Faculty of Public Health III, Lebanese University, Tripoli 1300, Lebanon; 6 Department of Medical Laboratory Sciences, Faculty of Health Sciences, University of Balamand, Beirut P.O. Box 55251 Sin El Fil, Lebanon

**Keywords:** *Jania rubens*, colorectal cancer, anticancer activity, epithelial-to-mesenchymal transition (EMT), TET enzymes, reactive oxygen species (ROS), epigenetic modulation

## Abstract

The red seaweed *Jania rubens* (*J. rubens*) is prevalent along the Lebanese coast and has drawn attention for its notable antineoplastic properties. Our previous data showed that its dichloromethane–methanol (DM) extract possesses antioxidant, cytotoxic, and anti-migratory effects on colon cancer cells. In the present study, a GC-MS analysis of DM extract identified a diverse profile of bioactive compounds, including flavonoids and pyrazole derivatives with antioxidant and anticancer activities. In vitro assays demonstrated that the DM extract exerts significant cytotoxic activity against various cancer cell lines, including colon, breast, and cervical types. Further investigation into the underlying molecular mechanisms revealed that the extract induces G2/M cell cycle arrest and reduces the expression of EMT (epithelial–mesenchymal transition) markers, N-cadherin and Twist. In addition, the extract showed anti-metastatic properties through its ability to decrease MMP-2 and MMP-9 activity. Mechanistically, DM caused a substantial reduction in Ten-Eleven Translocation (TET) enzymes *TET-1*, *TET-2*, and *TET-3*, which are essential DNA demethylation regulators, thus decreasing their enzymatic product 5-hydroxymethylcytosine (5-hmC). Interestingly, despite a significant increase in intracellular ROS (reactive oxygen species), suggesting a contribution to cytotoxicity, no substantial change in the biogenesis of promyelocytic leukemia nuclear bodies (PML-NBs) was detected. These findings demonstrate that *J. rubens* DM extract contains bioactive compounds with multiple anticancer effects, thus making it a promising candidate for developing new therapeutic agents.

## 1. Introduction

Colorectal cancer (CRC) is the second leading cause of cancer-related fatalities and the third most frequently diagnosed malignancy globally [1]. Sixty percent of cases are diagnosed between the ages of 50 and 74, whereas nearly 10% arise in those under 50 [2]. By 2040, CRC cases are expected to rise to 3.2 million additional cases and 1.6 million fatalities [2]. Lebanon has one of the highest cancer incidences in the Arab region [3,4]. Statistics showed that CRC is the third most prevalent cancer in both sexes, accounting for 5.8% of cancer-related mortality in the country [1]. Chemotherapy, surgery, radiation, or a combination of these for advanced-stage cancers are often the most common forms of treatment for the disease [5]. Traditional chemotherapeutic drugs prevent tumor growth and invasion but also adversely affect the environment of tumor cells [6]. In addition, some individuals may experience limited efficacy and develop drug resistance [7]. Thus, creating new, effective treatments for CRC is a continuous challenge. Statistics have shown that more than half of anticancer drugs originate from natural resources [8].

Tumor cells usually produce higher reactive oxygen species levels (ROS) than non-cancerous cells [9]. Increased ROS production is associated with cancer cell survival, tumor propagation, and invasion [9]. Recently, numerous studies have shown that ROS plays a dual role in cancer. Although high ROS levels are essential for tumor initiation and progression, the massive accumulation of ROS in cancer cells plays an anticarcinogenic role [10,11]. Excess accumulation of ROS can inhibit tumor cell growth and trigger apoptosis in cancer cells by activating ER stress [12,13,14], mitochondrial damage [15], P53-mediated apoptotic pathways [16,17], and the ferroptosis pathway in cancer [11]. In addition, the massive accumulation of ROS inhibits the EGF and EGFR-mediated cell proliferation pathways, causing cancer cell cycle arrest. The anticancer properties of ROS may offer an alternative approach for cancer therapy.

Another key player with a complex role in cancer is the Ten-Eleven Translocation (TET) family of enzymes. These epigenetic regulators induce DNA demethylation by converting 5-methylcytosine (5-mC) into 5-hydroxymethylcytosine (5-hmC) via a sequence of oxidation reactions [18]. The function of TET enzymes in cancer varies depending on tumor type and biological context. Loss-of-function mutations of *TET-2* have been frequently reported in hematological malignancies [19]. In addition, *TET-1* overexpression suppresses cell proliferation in human lung adenocarcinoma and hematopoietic malignancies [20,21]. Conversely, *TET-1* plays an oncogenic role in triple-negative breast cancer and MLL-rearranged leukemia [22,23]. Whereas overexpression of *TET-3* is associated with tumor progression [24], and its cell proliferative effect is believed to involve the activation of the PI3K/AKT/GSK3β/β-catenin pathway [25]. The diverse functions of TET enzymes in cancer label them as context-sensitive targets for therapy. Depending on the local cellular milieu and underlying molecular alterations, these enzymes can drive malignant growth or inhibit tumor progression. Systematic assessment of *TET* isoform expression and mutation patterns in patient-derived tumors can guide the tailoring of treatment; therapy may require selective inhibition of an overactive *TET* variant or, in opposite instances, the activation of a *TET* variant whose pathway is blunted. Such a dual facet of activity for the same family of enzymes illustrates why generic modulation is insufficient, and why precision oncology frameworks must integrate the functional context of each *TET* isoform on a tumor-by-tumor basis.

The urgent need for new CRC treatments that surpass existing limitations drives researchers to investigate natural marine organisms as a promising solution. Among natural aquatic resources, seaweed or macroalgae stand out as the most promising sources for discovering new bioactive compounds. These molecules demonstrate strong potential for developing nutraceuticals, pharmaceuticals, and pharmacological products [26]. The Red algae category comprises more than 7000 different species, forming an essential group. The active molecules found in red algae include polysaccharides together with proteins, unsaturated fatty acids, vitamins and minerals, phycobiliproteins, and additional compounds which are valued for their diverse applications in both biological and industrial fields [27]. In particular, red seaweed contains a significant concentration of phenolic compounds, such as flavonoids, which have various medical applications due to their interactions with proteins, including enzymes and cellular receptors [28]. Notably, seaweed-derived flavonoids are thought to possess unique chemical structures and bioactivities distinct from their terrestrial counterparts. These compounds act as potent antioxidants and have demonstrated the ability to induce apoptosis and autophagy, contributing to their anticancer potential [29].

The Lebanese coastline contains a rich variety of seaweed because it hosts more than 94 different red algal species from the Eastern Mediterranean Sea region. Research on *J. rubens* from the Mediterranean Sea has revealed multiple bioactive compounds which demonstrate anticancer properties [30,31,32].

In our previous study, we also demonstrated that *J. rubens* is rich in carbohydrates and proteins and that the red alga’s organic extract (dichloromethane–methanol; DM Soxhlet extract) has antioxidant, antiproliferative, and anti-migratory effects on colon cancer cells [33]. Despite these promising findings, studies elucidating the precise cellular pathways underlying *J. rubens*’s anticancer effects remain limited, and its overall antineoplastic potential necessitates further comprehensive examination. Therefore, this study aims to elucidate the molecular mechanisms responsible for the antiproliferative and anti-migratory properties of the *J. rubens* DM extract, specifically on HCT-116 human colon cancer cells.

## 2. Materials and Methods

### 2.1. Preparation of the Extract

Powdered algae are extracted with dichloromethane/methanol (DM, 1:1) at elevated temperature using a Soxhlet extractor for six cycles to prepare the DM Soxhlet extract. Then, the extract is concentrated using a rotary evaporator. The obtained solid extract is diluted with DMSO to a concentration of 100 mg/mL. An intermediate dilution of 1000 µg/mL is prepared using Dulbecco’s Modified Eagle Medium (DMEM, high glucose) (Sigma, St. Louis, MO, USA), followed by additional dilutions to achieve the final concentrations (100, 250, 500, 750 μg/mL).

DMSO concentration was maintained below 1%, and DMSO-only control was included to confirm that the observed effects were primarily attributable to the extract rather than the solvent. No significant cytotoxicity was observed in DMSO-treated wells at the concentrations used.

### 2.2. Cell Culture

Colon cancer cell lines (HCT-116, Caco-2), breast cancer cells (MDA-MB-231, MCF-7), and Hela were obtained from the American Type Culture Collection (ATCC, Manassas, VA, USA). Cells are cultured in Dulbecco’s Modified Eagle Medium (DMEM, high glucose) (Sigma, St. Louis, MO, USA) in a humidified incubator at 37 °C, with 5% CO_2_ and 95% air. Media is supplemented with 10% fetal bovine serum (FBS, Sigma-Aldrich, St. Louis, MO, USA) and 1% Penicillin–Streptomycin 100 IU/mL^−1^ (PS, Corning, Corning, NY, USA).

### 2.3. Cell Viability Assay

The cell viability assay is an MTT-based method that measures the ability of metabolically active cells to convert tetrazolium salt into formazan blue. Cells are seeded in a 96-well plate and left to adhere and reach 80% confluency. Cells are treated for 24 h with various DM concentrations, then incubated with MTT [3-(4, 5-dimethylthiazol-2-yl)-2, 5-diphenyltetrazolium bromide] (Sigma, St. Louis, MO, USA) for four hours at 37 °C in the dark. DMSO is added for 15 min before reading the absorbance. The ELISA microplate reader measured absorbances at 570 nm.

### 2.4. Crystal Violet Assay

*J. rubens* DM Soxhlet long-term cytotoxicity was assessed using a standard crystal violet assay. Briefly, HCT-116 cells (20,000) were seeded in 6 well-plate and left to adhere for 48 h; next, the cells were treated with different concentrations of DM Soxhlet (100, 250, 500, 750 μg/mL) for 24 h. The cells were then left to proliferate for 1 week. Finally, the cells were stained with 0.1% crystal violet (Sigma-Aldrich, St. Louis, MO, USA; Cat. No. 94448) diluted in PBS. The number of colonies is calculated using ImageJ software (version 1.54d, National Institutes of Health, Bethesda, MD, USA).

### 2.5. Cell Cycle Assay

Dead and living cells were collected 24 h after treatment with different concentrations of DM Soxhlet extract. The pellets were washed with cold PBS fixed with 70% cold ethanol and stored at −20 °C. For staining, cells were washed with cold PBS and incubated for one hour with 100 μL DNase-free RNase A (200 μg/mL) before staining with 1 mg/mL of propidium iodide (Sigma-Aldrich, St. Louis, MO, USA; Cat. No. P4170) for 15 min. The fluorescence intensity was measured by flow cytometry using a Guava EasyCyte8 flow cytometer.

### 2.6. Quantitative ROS Determination

Reactive oxygen species (ROS) were measured using the DCFDA/H2DCFDA Cellular ROS Assay Kit (Abcam, Cambridge, UK; Cat. No. ab113851) following the manufacturer’s instructions. Briefly, HCT-116 cells were seeded in a clear-bottom, dark 96-well microplate and allowed to attach overnight. The following day, the culture medium was aspirated carefully, and 100 µL of 1× buffer was added to and discarded from each well to wash the cells. Then, 100 µL of 20 µM DCFDA solution was added to each well for staining, and the plate was incubated at 37 °C for 45 min in the dark. After incubation, the DCFDA solution was removed, and 100 µL of 1× supplemented buffer with 10% FBS without phenol red was added. The cells were then left untreated or treated with 100 µL of *J. rubens* DM extract at the final concentrations of 250 or 500 µg/mL and incubated for two hours without washing according to instructions to preserve intracellular fluorescence. Finally, ROS levels were quantified immediately using a fluorescence plate reader (excitation/emission: 485/535 nm).

### 2.7. Immunofluorescence Assay

HCT-116 cells were seeded on coverslips in 24-well plates, adhering to and proliferating to 70–80% confluency. HCT-116 cells were then treated with DM Soxhlet extract for four, eight and twelve hours. Cells were fixed with 4% formaldehyde for 20 min and blocked for 1 h with a 5% BSA solution. Then, cells were incubated overnight with the following antibodies (rabbit anti-PML 1:500) (rabbit anti-TET-1 1:500 Gene Tex, Irvine, CA, USA; Cat. No. GTX124207) (rat anti-5hmC Diagenode Liège, Belgium; Cat. No. C1522001). Next, cells were incubated with Texas red anti-rabbit, fluorescein anti-rabbit, or fluorescein anti-rat conjugated secondary antibody for one hour, followed by DAPI nuclear staining for 10 min. Finally, cells were mounted using an antifade reagent (Abcam, Cambridge, UK; Cat. No. ab104135). Images were processed and analyzed with confocal microscopy integrated with ZEN Imaging Software-LSM710. The ImageJ software was used for post-confocal quantification.

### 2.8. Zymography

Cells were cultured in T75 flasks to 80% confluency, then treated with DM extract (250 or 500 µg/mL) or left untreated for 24 h, followed by incubation for an additional 24 h in fresh incomplete Dulbecco’s Modified Eagle’s Medium (DMEM, high glucose; Sigma, St. Louis, MO, USA). Supernatants were collected and concentrated using a dialysis membrane (Spectra/Por tubing, 6–8 kDa; Spectrum Laboratories, Rancho Dominguez, CA, USA) and poly (ethylene glycol) (PEG, 35,000; Sigma-Aldrich, St. Louis, MO, USA; Cat. No. BCCJ5533). Protein concentrations were determined with the DC Protein Assay II kit (Bio-Rad, Feldkirchen, Germany). Equal amounts of protein (30 µg) were resolved on 10% SDS–PAGE gelatin gels at 90 V for 3 h 30 min in running buffer (Tris, glycine, SDS, pH 8.3). 1% BSA was used as a positive control for MMP-2 and MMP-9, and Precision Plus Protein™ Kaleidoscope™ Prestained Protein Standards (Bio-Rad, Hercules, CA, USA, Cat. No. 1610375) served as the molecular weight marker. Gels were incubated overnight at 37 °C in substrate buffer containing 50 mM Tris-HCl (pH 8.0), 6.6 mM calcium chloride (anhydrous), and 3.1 mM sodium azide. They were then stained with Coomassie Brilliant Blue R-250 (Bio-Rad, Hercules, CA, USA) for 1 h, and destained (methanol, acetic acid, water) until clear bands appeared. Images were acquired using the ChemiDoc™ Imaging System (Bio-Rad, Hercules, CA, USA), and band intensity quantified with ImageJ software.

### 2.9. Analysis with Gas Chromatography-Mass Spectrometry (GC-MS)

This study was conducted utilizing an Agilent Technologies gas chromatograph outfitted with a mass selective detector, HP-5MS (Agilent, Beijing, China). A 5% phenyl methyl siloxane capillary column of 30.0 m × 250 μm × 0.25 μm was employed, with helium as the carrier gas at a flow rate of 1 mL/min. The column temperature was set to commence at 90 °C for 1 min, then increasing at a rate of 8 °C/min to 205 °C, then at 5 °C/min to 240 °C, and finally at 8 °C/min to 300 °C. The mass spectrometer was operated at 70 eV. The contents were identified by comparing their mass spectrum data with reference compounds from the National Institute of Standards and Technology (NIST) spectrum Library.

### 2.10. RNA Extraction, Reverse Transcription, and qRTPCR

Total RNA was extracted using TRIzol reagent, followed by chloroform-induced phase separation and centrifugation at 12,000 rpm for 15 min at 4 °C. The RNA-containing supernatant was collected and precipitated with isopropanol, then centrifuged. The resulting RNA pellet was washed twice with 75% ethanol at 7500 rpm and air-dried at room temperature for 20 min. 20 μL of nuclease-free water was then added to the pellet. Finally, RNA was quantified using a Nanodrop spectrophotometer (ND-1000 Spectrophotometer, Thermo Fisher Scientific, Wilmington, NC, USA), and the samples were stored at −20 °C.

1 μg of RNA was reverse-transcribed to cDNA using Iscript cDNA Synthesis Kit (Bio-Rad Laboratories).

Gene expression analysis. Quantitative real-time polymerase chain reaction (qRTPCR) was performed in a CFX96™ Real-Time PCR Detection System (Bio-Rad, Hercules, CA, USA) using the qPCR Sybr Green Master Mix. The relative fold change in gene expression was calculated using the ΔΔ^Cq^ method after normalization to the housekeeping gene GAPDH. The setting of PCR was outlined as follows: A pre-cycle at 95 °C for 5 min followed by 40 cycles consisting of 95 °C for 10 s, 60 or 57–58 °C (according to the gene tested) for 30 s, and 72 °C for 30 s with a final extra-elongation at 72 °C for 5 min (Table 1).

### 2.11. Statistical Analysis

All statistical analyses (unpaired *t*-test, one-way ANOVA, and two-way ANOVA) were performed using GraphPad Prism 10 (version 10.0; GraphPad Software, Boston, MA, USA). Probability values below 0.05 (* *p* < 0.05) are considered significant, and values below 0.01 (** *p* < 0.01), 0.001 (*** *p* < 0.001), and 0.0001 (**** *p* < 0.0001) are considered highly significant. The quantitative data are expressed as means ± SD from the indicated set of experiments.

## 3. Results

### 3.1. Major Bioactive Compounds Identified in Jania rubens DM Extract

The GC-MS analysis of *J. rubens* DM extract revealed a wide range of bioactive secondary metabolites. The identified compounds in the extract show biological significance and therapeutic value, especially for cancer research applications. The major compounds found in the extract were flavonoid derivatives such as 4′-Methoxyflavone, 7-Methoxyflavone, 4H-1-Benzopyran-4-one, 8-methoxy-2 -phenyl- (also known as 8-methoxyflavone) and 2-[3-Methoxyphenyl]-4H-1-benzopyran-4-one, which had high match quality and relatively high peak areas. Pyrazole-based compounds were also detected, including 5-amino-1,3-diphenyl-1H-pyrazole, 1-(Anilinomethylene)-2-indanone, and Indan-1,3-dione, 2-(1,3-dimethyl-1H-pyrazol-4-ylmethylene). Additionally, heterocyclic and nitrogen-containing compounds such as 4-[5-(4-Methoxyphenyl)-2-oxazolyl] pyridine and Oxazolo [3,2-e] xanthine were identified. Other constituents such as 4-bromo-N-(2-chloroacetyl) pyrazole-1-carboxamide and 2,3-dihydro-2-hydroxymethyl-5,7-dimethyl-4H-1-benzopyran-4-one were also detected. The retention times of these compounds ranged from 27.09 to 51.63 min, and their chemical identities were supported by CAS number matching and high library search quality scores. The detailed results are summarized in Table 2 and Figure S1.

### 3.2. J. rubens DM Extract Decreases the Viability of Various Solid Cancer Cell Lines

**Figure 1:**
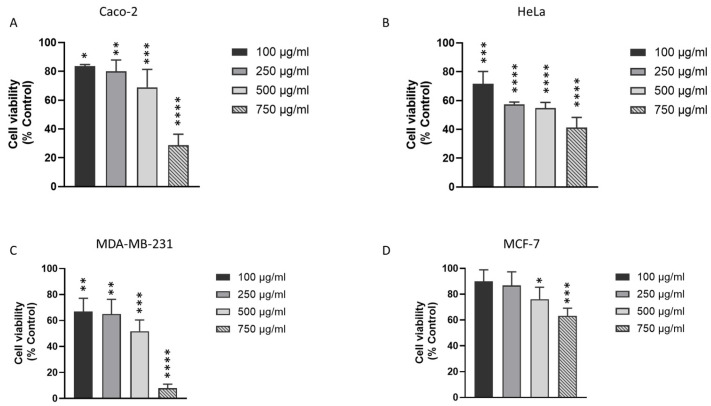
Effect of DM Soxhlet extract on the viability of different solid cancer cell lines. MTT assay was used to assess the viability of Caco-2 colon cancer cells, MCF-7 and MDA-MB-231 breast cancer cells, as well as HeLa cervical cancer cells after 24 h of treatment with various concentrations of the DM Soxhlet extract (0-100-250-500-750 µg/mL). (**A**) Caco-2, (**B**) HeLa, (**C**) MDA-MB-231, and (**D**) MCF-7 cells treated with DM Soxhlet extract for 24 h. The percentage of cell viability was calculated considering the value of the control as 100%. Results are presented as the mean ± SD (n ≥ 3). * *p* < 0.05, ** *p* < 0.01, *** *p* < 0.001, **** *p* < 0.0001 vs. control group. DM: Dichloromethane/Methanol.

We previously demonstrated that *J. rubens* DM extract induces a significant cytotoxic effect against HCT-116 and HT-29 colon cancer cell lines with IC_50_ of 499.94 µg/mL and 531.44 µg/mL, respectively. To study the effect of DM anticancer effect on other solid cancer cells, we treated Caco-2 colon cancer cells, MCF-7, and MDA-MB-231 breast cancer cells, in addition to HeLa cervical cancer cells, with various concentrations of DM Soxhlet extract (100-250-500-750 µg/mL) for 24 h. MTT results showed that the viability of the four cell lines decreased in a dose-dependent manner (Figure 1). At 24 h, treatment with 100 µg/mL DM Soxhlet extract reduced Caco-2 cell viability to 82%, and further to 35% at 750 µg/mL. In HeLa cells, viability decreased to 72% at 100 µg/mL and 42% at 750 µg/mL. Similarly, MDA-MB-231 cell viability was reduced to 67% at 100 µg/mL and 8% at 750 µg/mL, while MCF-7 cells showed a reduction to 92% at 100 µg/mL and 63% at 750 µg/mL. Further analysis indicated that MCF-7 cells were the most resistant to DM treatment among the tested cell lines, with an IC_50_ of 1165.25 µg/mL. In contrast, the IC_50_ values for MDA-MB-231, Caco-2, and HeLa cells were 437.57 µg/mL, 628.97 µg/mL, and 641 µg/mL, respectively.

### 3.3. J. rubens DM Extract Induced Cell Cycle Arrest and Inhibited the Long-Term Survival of HCT-116 Cells

**Figure 2:**
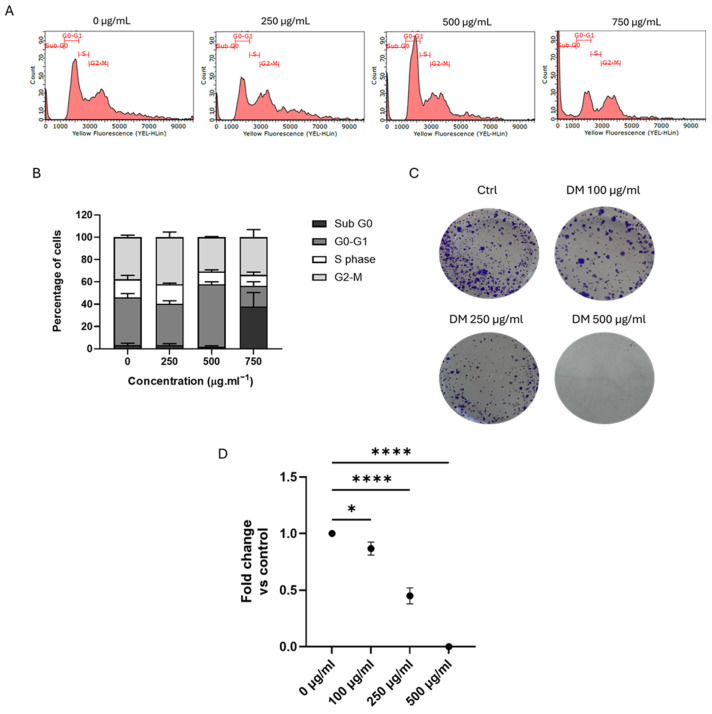
Effect of *J. rubens* DM Soxhlet extract on cell cycle progression and long-term survival of HCT-116 cells. (**A**) Representative flow cytometry histograms. Peaks correspond to different phases of the cell cycle (Sub G0, G0–G1, S, and G2–M). (**B**) Quantification of cell cycle phase distribution based on flow cytometry analysis. (**C**) Representative images of crystal violet assay showing the effect of increasing concentrations of DM extract (0, 100, 250, and 500 μg/mL) on HCT-116 cell survival. (**D**) Colony numbers are quantifiable as fold change relative to the untreated control (0 μg/mL). Data values are represented as mean ± SD (n = 3). * *p* < 0.05, **** *p* < 0.0001.

To investigate the molecular pathways underlying the anticancer effects of DM extract, we used HCT-116 colon cancer cells as a model system for detailed analysis. To investigate the cytotoxic mechanism of DM Soxhlet on HCT-116 cells, we performed cell cycle analysis on HCT-116 cells using flow cytometry (Figure 2). DM Soxhlet extract induced significant changes in the cell cycle of HCT-116 cells. Untreated cells presented a typical cytogram of a diploid cell population. Treated cells with 500 μg/mL of DM Soxhlet extract for 24 h demonstrated a significant accumulation in the G0–G1 phase, increasing by 43.7% compared to 32.66% for the control. Moreover, the number of cells in the sub-G0 phase increased considerably upon treatment with 750 μg/mL, reaching 29.78% compared to 2.58% in control (Figure 2A,B). This notable increase in the sub-G0 phase strongly suggests an induction of apoptosis, as deregulation of the cell cycle is closely associated with programmed cell death.

In a previous study, we demonstrated a dose-dependent decrease in HCT-116 cell viability following treatment with the DM extract for 24 and 48 h [33]. To assess the long-term survival and self-renewal capacity of HCT-116 cancer cells following DM extract treatment, cells were exposed to various extract concentrations for 24 h and then allowed to grow for one week. Crystal violet staining revealed a clear, dose-dependent reduction in both the size and number of colonies. At 100 μg/mL, the colonies decreased slightly to 0.85-fold relative to the untreated control, indicating an initial inhibitory effect. This reduction became significantly more pronounced at 250 μg/mL, with colony numbers declining dramatically to an average of 0.45-fold. At 500 μg/mL, colonies were eliminated. These results suggest that cells experiencing DM-induced cell cycle arrest could not re-enter the cell cycle, ultimately leading to a profound inhibition of clonogenic survival and subsequent cell death.

### 3.4. J. rubens DM Extract Downregulates EMT Markers and Inhibits Matrix Metalloproteinase Activity in HCT-116 Cells

**Figure 3:**
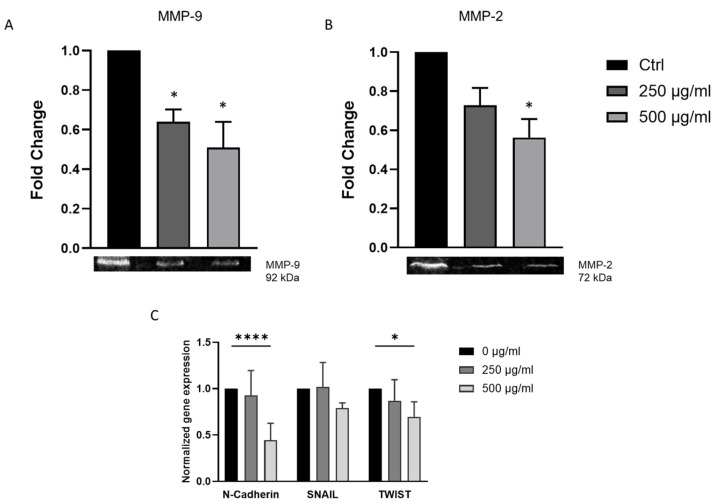
Analysis of EMT markers expression and matrix metalloproteinase activity in HCT-116 cells after exposure to *J. rubens* DM Soxhlet extract. (**A**,**B**) Gelatin zymography analysis of HCT-116 cells treated with 250 and 500 μg/mL DM extract for 24 h. Proteins were resolved on a gelatin-containing polyacrylamide gel to evaluate MMP-9 and MMP-2 enzymatic activity. The bar graph shows the densitometric quantification of zymographic bands. Data represents results from two independent experiments. * *p* < 0.05. (**C**) mRNA expression levels of EMT-related genes (N-cadherin, TWIST, and SNAIL) in HCT-116 cells treated with 250 and 500 μg/mL DM extract for 16 h. Gene expression was quantified by qRT-PCR, normalized to GAPDH, and presented relative to untreated control cells. Bar graphs represent the mean ± SD from three to five independent experiments. * *p* < 0.05, **** *p* < 0.0001.

Epithelial-to-mesenchymal transition (EMT) is closely associated with the increased metastatic potential of cancer cells. To investigate the mechanisms underlying the anti-migratory effects of DM extract treatment, we examined whether DM treatment alters the expression of key EMT markers. Transcriptional levels of N-cadherin, SNAIL, and TWIST were analyzed using RT-PCR. The results revealed that treatment with 500 µg/mL DM significantly reduced the expression of N-cadherin and TWIST by 0.4- and 0.7-fold, respectively, compared to the control. However, no significant change was observed in SNAIL expression (Figure 3C).

Matrix metalloproteinases (MMPs) are essential for cancer cells invasion. MMP-9 is especially important in the initiation and growth of CRC. High levels of MMP-9 are strongly linked to more tumor invasion, lymph node metastasis, and poor prognosis in CRC patients. This makes it a useful negative prognostic marker [34,35]. Multivariate analyses have also shown that MMP-2 expression is an independent predictor of survival. High MMP-2 expression is strongly linked to advanced tumor stage, lymph node involvement, and distant metastasis. It is also strongly linked to lower overall survival [36,37].

Due to the recognized functions of MMP-2 and MMP-9 in extracellular matrix degradation and colorectal cancer progression, these gelatinases were chosen for examination. The supernatants from HCT-116 cultures, gathered and concentrated post-treatment with *J. rubens* DM Soxhlet extract (250 and 500 µg/mL), were directly analyzed using gelatin zymography without undergoing purification. By comparing them to molecular weight standards, we found bands that matched MMP-2 (72 kDa) and MMP-9 (92 kDa). A significant, dose-dependent reduction in MMP-9 activity was observed following 24 h of DM treatment. In contrast, MMP-2 activity was significantly reduced only at the 500 µg/mL concentration (Figure 3A,B).

### 3.5. J. rubens DM Soxhlet Extract Increases ROS Production in HCT-116 Cells

**Figure 4:**
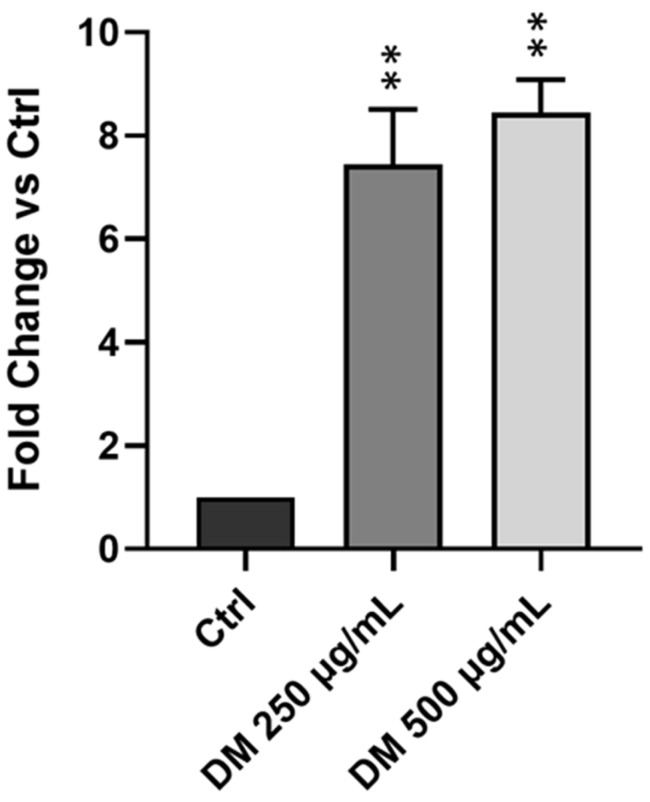
Effect of *J. rubens* DM Soxhlet extract on ROS production in HCT-116 cells. HCT-116 cells were cultured as control (Ctrl) or treated for two hours with 250 or 500 μg/mL DM extract. ROS levels were quantified using DCFDA assay. Results values were expressed as fold change relative to the untreated control and represented as mean ± SD from two independent experiments (n = 2), each performed with four replicates. ** *p* < 0.01.

Many natural source-derived antioxidants identified as effective against cancer exert their anticancer effects primarily by inducing excessive levels of ROS within cancer cells [38]. We previously demonstrated that the DM Soxhlet extract possesses antioxidant and anticancer properties [33]. To determine ROS production, we performed a DCFDA-based fluorescence assay. *J. rubens* DM extract resulted in a marked increase in intracellular ROS production in HCT-116 cells. As shown in Figure 4, treatment with 250 µg/mL DM resulted in an approximately 7-fold increase of the ROS level, and 500 µg/mL induced an 8-fold increase compared to the control. No significant difference was observed between the two concentrations used. The findings show that the DM extract remarkably induces ROS generation in HCT-116 cells, supporting its capacity to induce oxidative stress–mediated cytotoxicity.

### 3.6. J. rubens DM Extract Decreases TET-1, TET-2, TET-3 and 5-hmC Expression

**Figure 5:**
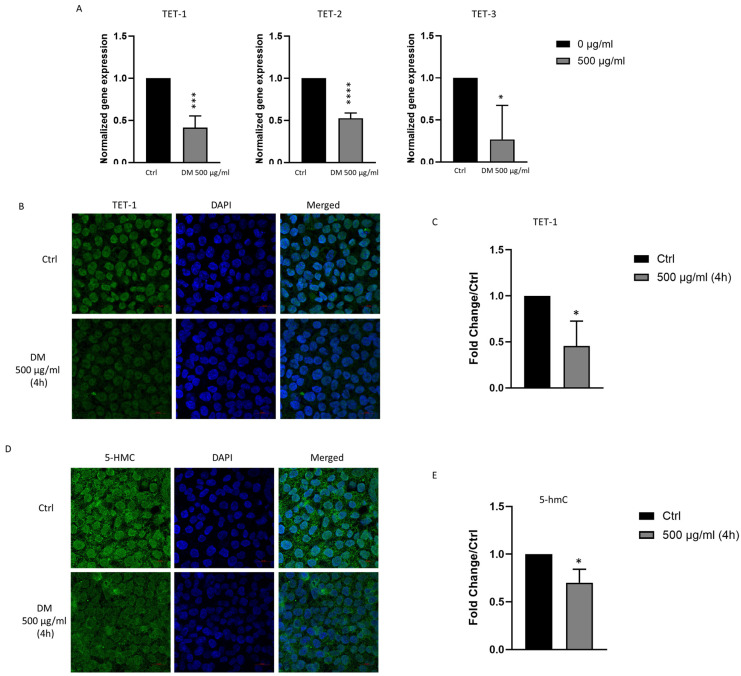
Assessment of *J. rubens* DM Soxhlet extract on the expression of *TET-1*, *TET-2*, *TET-3*, and 5-hmC. (**A**) mRNA expression levels of *TET-1*, *TET-2*, and *TET-3* in HCT-116 cells treated and untreated with 500 μg/mL DM extract for 16 h. Gene expression was quantified by qRT-PCR, normalized to GAPDH, and presented relative to untreated control cells. Results are presented as the mean ± SD from three to four independent experiments. For immunofluorescence staining, HCT-116 was cultured untreated or treated for four hours. (**B**) Demonstrative microscopic images for TET-1 expression in HCT-116 cells. Anti-TET-1 antibody was used to mark TET-1 (green), while DAPI stained the nucleus with blue (63× oil). (**C**) Fluorescence intensity was measured by ZEN software and normalized to the control. (**D**) Demonstrative microscopic images for 5-hmC expression in HCT-116 cells. Anti-5-hmC antibody was used to mark 5-hmC (green), while DAPI stained the nucleus with blue (63× oil). (**E**) Fluorescence intensity was measured by ZEN software and normalized to the control. Data presented as the mean ± SD from two to four independent experiments. * *p* < 0.05, *** *p* < 0.001, **** *p* < 0.0001. Scale bar 20 μm.

TET enzymes play a complex role in cancer progression; while some studies have demonstrated their tumor-suppressive functions [39], others have suggested potential oncogenic activity [22]. To investigate whether DM extract affects the expression and function of TET enzymes, we evaluated both *TET* expression and total 5-hmC levels in HCT-116 cells following treatment. HCT-116 cells were treated with and without 500 μg/mL DM extract, and RT-PCR was used to evaluate the transcriptional levels of *TET-1*, *TET-2*, and *TET-3*. The results demonstrated a significant decrease in the mRNA expression of all three enzymes following treatment (Figure 5A). Additionally, immunofluorescence staining was used to examine TET-1 protein expression and total 5-hmC content. Consistent with the RT-PCR results, the immunofluorescence analysis indicated that TET-1 protein and 5-hmC levels significantly decreased in DM-treated HCT-116 cells (Figure 5B–E). These findings suggest that DM extract inhibits DNA demethylation pathways by decreasing TET enzyme expression and activity, thereby indicating that the anticancer activity of DM extract may be mediated by altering the DNA methylation process.

### 3.7. J. rubens DM Extract Does Not Affect PML Nuclear Body Biogenesis

**Figure 6:**
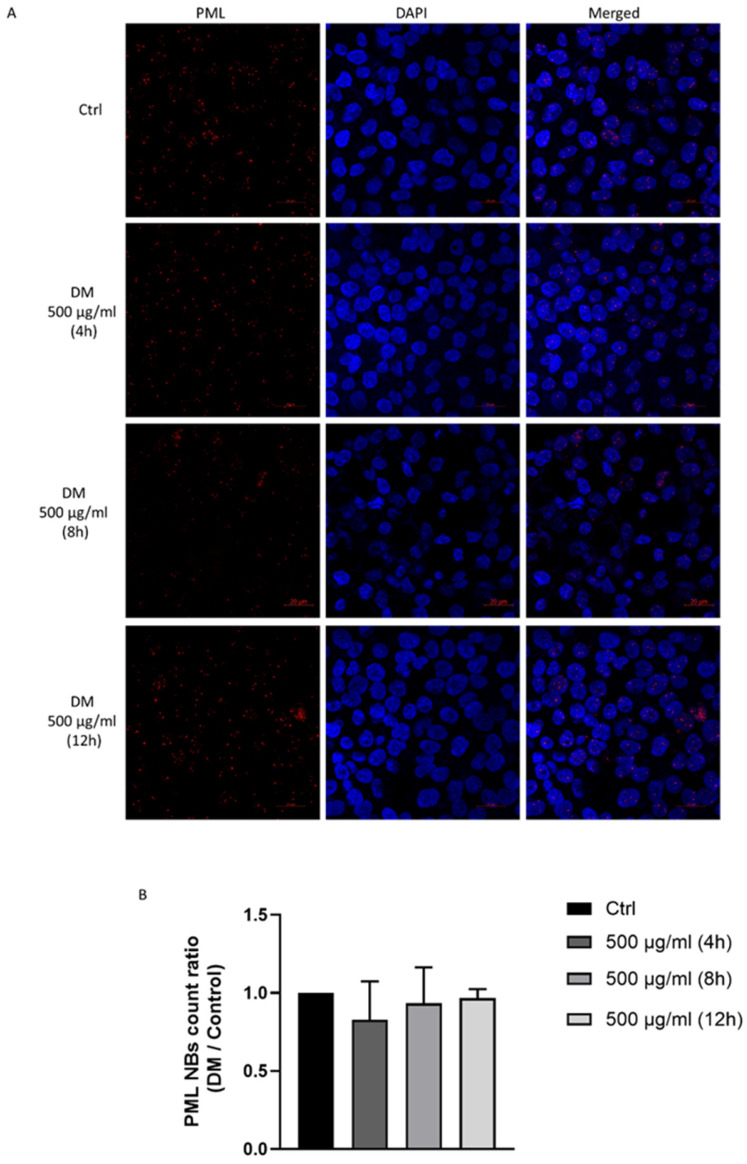
Analysis of PML nuclear body biogenesis in HCT-116 cells following treatment with *J. rubens* DM Soxhlet extract. HCT-116 cells were either left untreated (control) or treated with 500 μg/mL DM extract for 4, 8, or 12 h. (**A**) Representative fluorescence microscopy images showing PML expression (red) using anti-PML antibody, with nuclei counterstained using DAPI (blue). (63× oil) (**B**) Quantification of PML nuclear bodies per nucleus expressed as fold change relative to control. Data represent the mean from approximately 250 cells per condition.

Promyelocytic leukemia nuclear bodies (PML-NBs) are nuclear structures associated with transcriptional regulation, viral infection response, genomic stability, apoptosis induction, and tumor suppression. Promyelocytic leukemia protein (PML) has been identified as a sensor of ROS, responding to oxidative stress by activating p53 and promoting Nrf2-dependent antioxidant responses [40,41]. To explore PML-NBs biogenesis in response to DM extract, we treated HCT-116 with 500 μg/mL for four hours, eight hours, and 12 h. No evident changes were detected in the number, size, or distribution of PML- NBs at any of the tested time points following DM extract treatment compared to the untreated control group (Figure 6). This indicates that *J. rubens* DM ROS production does not trigger PML-associated stress responses.

## 4. Discussion

This study offers new mechanistic insights into the anticancer activity of *J. rubens* dichloromethane–methanol (DM) extract against HCT-116 colon cancer cells. The study explained the molecular basis of its antitumor properties and identified the extract’s chemical makeup through GC-MS analysis, which showed multiple bioactive compounds with established anticancer and antioxidant effects. The research builds upon our previous findings, which showed the extract’s antioxidant properties, cytotoxic effects, and anti-migratory capabilities by establishing connections between its observed bioactivity and its molecular effects and chemical constituents [33,42].

A principal observation in the current study is the induction of cell cycle arrest in HCT-116 cells after exposure to *J. rubens* DM extract. Cell cycle dysregulation is a hallmark of cancer, and disrupting cell cycle checkpoints remains a promising strategy in anticancer therapies [43]. Several cell cycle proteins, including D- and E-type cyclins, CDKs (CDK2, CDK4, CDK6), PLK1, and Aurora kinases, are commonly overexpressed in cancers and contribute to tumor development and progression [43]. The checkpoint kinases CHK1 and WEE1 represent potential drug targets for treating cancers without functional p53 [44,45,46]. CDK4/6 inhibitors, including palbociclib, ribociclib, and abemaciclib, have proven highly effective in treating estrogen receptor-positive HER2-negative breast cancer [47]. PLK1 inhibitors, such as rigosertib and volasertib, in combination with the Aurora A inhibitor alisertib, have shown beneficial effects in treating hematologic malignancies and solid tumors [43,48].

The extract’s ability to inhibit cell cycle progression is likely associated with its previously described cytotoxic activities [33]. This finding confirms that the extract affects the essential regulatory proteins of the cell cycle, leading to the inhibition of cancerous cell proliferation. Cell cycle arrest is a common mode of action for naturally occurring molecules with cytostatic or cytotoxic activities, which are often followed by subsequent processes such as apoptosis and senescence [49,50]. While the identification of specific interrupted checkpoints requires further study, our findings align with existing evidence that demonstrates the ability of seaweed-derived bioactive compounds to interfere with the progression of the cell cycle in various cancer models [51,52,53].

Along with its ability to inhibit cell proliferation, the extract exhibited anti-metastatic activity by significantly downregulating the expression of key epithelial-to-mesenchymal transition (EMT) markers, specifically N-cadherin and Twist, as well as by reducing the enzymatic activities of MMP-2 and MMP-9. EMT is a crucial process by which epithelial cancer cells acquire mesenchymal features, thereby enhancing their migratory ability and invasive capacity within tissues [54]. EMT is linked to several tumor functions, including tumor initiation, cancer progression, tumor stemness, cell migration, intravasation into the bloodstream, metastasis, and therapeutic resistance [55]. Twist is a crucial EMT transcription factor; its upregulation is associated with invasiveness, metastasis, and a poor prognosis in CRC [56]. N-cadherin is a mesenchymal marker and is correlated with metastasis and decreased survival rates among CRC patients [57]. The downregulation of these markers by the extract suggests the potential of *J. rubens* DM extract in reducing the metastatic capacity of cancer cells. MMPs are proteolytic enzymes that degrade elements of the extracellular matrix (ECM); they play a crucial role in cancer development by promoting tumor growth and metastasis [58]. The observed inhibition of MMP-2 and MMP-9 activity by the *J. rubens* DM extract provides further evidence of its role in suppressing tissue invasion and metastasis. These findings are consistent with previous studies showing that other seaweed active extracts, such as fucoidan, also inhibit EMT by suppressing mesenchymal markers and decreasing matrix metalloproteinase activity [59,60,61].

A notable finding in this study is the reduced expression of Ten-Eleven Translocation (TET) enzymes *TET-1*, *TET-2*, and *TET-3* and the decrease in their derivative, 5-hydroxymethylcytosine (5-hmC). TET enzymes play a crucial role in maintaining DNA demethylation, and their dysregulation has been linked to cancer development [62]. Furthermore, the expression levels of TET family genes are known to influence the clinical staging of cancer patients, their sensitivity to chemotherapeutic treatments, and their prognostic outcomes. This occurs through their regulation of the tumor microenvironment, cellular stemness capacity, and immune subtypes [63]. The observed decrease in TET enzyme levels suggests that the *J. rubens* DM extract may induce hypermethylation and subsequent gene silencing of oncogenes and other cancer-related pathways, thereby influencing the behavior of cancer cells. Given that global DNA hypomethylation is common in many cancers [64,65,66], the extracts’ ability to reverse this epigenetic alteration could represent a potent mechanism contributing to its antineoplastic activity. Future experiments should investigate the specific methylation alterations that occur in response to *J. rubens* DM treatment to fully understand this phenomenon. Together, these observations suggest that *J. rubens* is an epigenetic modifier that modulates gene expression while also displaying cytotoxic activity.

The extract produces substantial increases in ROS within cells, which serve as a major factor contributing to its toxic effects. The elevated production of ROS has the potential to create oxidative stress, resulting in DNA lesions and protein oxidation, which can ultimately lead to apoptosis in vulnerable cancer cells [67,68]. The high ROS levels generated by the extract did not result in noticeable changes to promyelocytic leukemia nuclear body (PML-NB) formation. These prominent nuclear structures play a critical role in various cellular processes, including tumor suppression, DNA repair, and apoptosis [69]. The unexpected observation indicates that the extract’s ROS-mediated cytotoxic effect operates through PML-independent pathways, which demonstrate different molecular mechanisms for its anticancer action. The PML-independent ROS effect results from multiple factors. PML NB formation occurs through a tightly controlled process which needs specific ROS concentrations or types to become activated [70]. The ROS levels produced by *J. rubens* DM extract may cause general oxidative damage and cytotoxicity but remain below the threshold needed to impact PML biogenesis [40,71]. Additionally, the extract’s ROS production leads to cytotoxic effects that occur before PML-mediated responses can be initiated. The DNA damage response triggered by ROS can activate cell cycle checkpoints and apoptotic pathways through ATM and ATR kinases, which lead directly to cell death without PML involvement [72]. This multifaceted action could explain the observed dissociation between significant ROS production and the lack of a PML nuclear body response.

GC-MS analysis of the *J. rubens* DM extract confirmed its biological potential by identifying several pharmacologically active compounds. The antioxidant and anti-proliferative effects of the extract are supported by the detection of flavonoids such as 4′-Methoxyflavone and 7-Methoxyflavone, both of which are known to modulate reactive oxygen species (ROS) levels, induce apoptosis, and disrupt cancer cell cycle progression. Notably, 7-Methoxyflavone and its derivatives have demonstrated anti-inflammatory effects [73], antioxidant effects [74], and anticancer properties [75,76]. Similarly, 4′-Methoxyflavone, a flavone derivative, has been reported to exhibit neuroprotective [77], antioxidant [78] and anticancer effects [79].

Flavone derivatives are known to exert cytotoxic effects across various cancer cell types. For example, apigenin induces apoptosis in MCF-7 breast cancer cells by increasing ROS production and promoting DNA fragmentation. It also enhances the expression of p53, increases the Bax/Bcl-2 ratio, activates caspases, and promotes PARP cleavage, ultimately causing cell cycle arrest at the G2/M phase [80]. Rutin exerts its cytotoxic effect by elevating ROS levels, leading to oxidative stress and apoptosis. It also inhibits the PI3K/Akt and Ras/Raf/MAPK signaling pathways, causing cell cycle arrest. Additionally, rutin modulates apoptosis-related proteins by upregulating Bax and downregulating Bcl-2 and MMP-2, activating caspase-3 [81].

Another well-studied flavonoid, quercetin, has been shown to arrest the cell cycle via p53-mediated pathways. It also inhibits cancer cell migration by upregulating E-cadherin and downregulating N-cadherin, Vimentin, MMP-2, MMP-7, and Snail, primarily inhibiting the Akt signaling pathway. Furthermore, quercetin can induce autophagic cell death by modulation of LC3-I expression [82].

The pyrazole derivative 5-amino-1,3-diphenyl-1H-pyrazole is recognized as an antimicrobial agent [83] with additional anticancer potentials [84,85]. Pyrazole-based compounds exhibit a diverse array of biological actions, covering antimicrobial, anticancer, anti-inflammatory, and antifungal effects [86,87]. Mechanistically, the pyrazole derivatives function through molecular mechanisms that target multiple cancer cell signaling molecules, including EGFR (epidermal growth factor receptor), BRAF V600E (B-Raf proto-oncogene, serine/threonine kinase), telomerase, Aurora-A kinase, and multiple receptor tyrosine kinases. The compounds can stop cancer cell growth, causing cell cycle arrest, apoptosis, and autophagy modulation [88]. In vitro studies demonstrate that pyrazoles show practical antiproliferative effects against different cancer cell lines, with HCT-116 colorectal carcinoma cells being among the most responsive [89].

The detection of 1-(Anilinomethylene)-2-indanone further contributes to the extract’s pharmacological significance. This compound is a derivative of indanone, a structure commonly encountered in pharmaceutical compounds. Indanones often demonstrate a variety of biological actions, including anti-Alzheimer’s, anticancer, antibacterial, and antiviral properties [90]. Furthermore, the benzopyran derivative 2-[3-Methoxyphenyl]-4H-1-benzopyran-4-one, is recognized for its diverse bioactive qualities, including antioxidant, anti-inflammatory, and anticancer effects [91,92].

The observed bioactivities of the extract receive additional functional support from compounds that contain oxazole and xanthine backbones. These chemical classes function as essential regulators of oxidative stress and epigenetic processes [93,94,95,96]. Certain oxazole derivatives can stop cell cycle progression [97]. The compounds work through two mechanisms: they block STAT3, which controls cancer cell survival and proliferation, or bind to tubulin to disrupt microtubule formation and trigger apoptosis [98]. Certain oxazoles function as protein kinase inhibitors that control fundamental cellular processes, including growth and cell division [98].

Although the extract was found to significantly reduce TET enzyme expression, a direct link between specific compounds and TET inhibition has not yet been established. To date, there is limited evidence in the literature directly connecting any of the compounds identified in our extract to TET enzyme inhibition. Further studies are needed to identify the specific components responsible for modulating the DNA demethylation pathway. However, certain polyphenolic compounds have been reported to modulate epigenetic mechanisms, including the activity of DNA methyltransferases (DNMTs) and the regulation of *TET* gene expression [99,100]. For example, the flavan-3-ol epigallocatechin-3-gallate (EGCG), the predominant flavonoid in green tea, has been shown to inhibit DNMT activity through direct enzyme interaction, resulting in decreased cellular levels of 5-methylcytosine [101,102]. Similarly, the flavonols quercetin, fisetin, and myricetin have demonstrated concentration-dependent inhibition of DNMT1-mediated DNA methylation [101]. Additionally, the flavones apigenin and luteolin have been shown to inhibit DNMT activity in KYSE-510 esophageal squamous carcinoma cells [103]. Another investigation reported that treatment with a standardized, bioavailable polyphenolic preparation (BDPP) markedly decreased the mRNA expression of *DNMT1*, *DNMT3A*, *DNMT3B*, *TET2*, and *TET3* while significantly increasing *TET1* expression in the hippocampus compared to the control group [99]. These changes suggest that BDPP may induce genome-wide alterations in DNA methylation by modifying the balance between DNMTs and TETs. Accordingly, BDPP treatment resulted in hypermethylation of nine genes and hypomethylation of six genes in the hippocampus [99].

The synergy among these classes of compounds may explain the extract’s multifaceted mechanism of action. These insights warrant future studies focused on fractionation and structural characterization of the DM extract to isolate and validate the active components.

In summary, this study offers a detailed elucidation of the molecular mechanisms through which the *J. rubens* DM extract exerts its anticancer effects in HCT-116 colon cancer cells. The extract functions as a multi-targeted agent that causes cell cycle arrest and suppresses metastasis by reducing EMT markers and MMPs while modifying epigenetic patterns by blocking TET enzymes and lowering 5-hmC content. The extract causes intracellular ROS accumulation, which results in cytotoxic effects through a PML-independent DNA damage response pathway. The GC-MS analysis detected multiple bioactive compounds, including flavonoids and pyrazole derivatives, which appear to generate the observed biological responses. The study demonstrates that *J. rubens* DM extract shows potential as a multi-functional anticancer agent, which warrants further clinical investigation.

## 5. Conclusions

The current study emphasizes the significant anticancer efficacy of the dichloromethane–methanol extract of *J. rubens*. The extract demonstrated broad-spectrum anticancer effects by inducing cell cycle arrest, inhibiting epithelial–mesenchymal transition (EMT) markers (N-cadherin and TWIST), suppressing MMP-2 and MMP-9 activity, downregulating TET enzyme expression and functional outputs, and augmenting ROS generation. These findings collectively elucidate the mechanisms underlying its cytotoxic and anti-metastatic effects in the HCT-116 CRC cell line. Due to its wide range of bioactive compounds, as found by GC-MS, *J. rubens* DM extract may represent a potential adjuvant in chemotherapy.

## Figures and Tables

**Table 1:** List of primers.

Gene	Primer Sequence	Annealing Temperature
N-Cadherin	F: 5′-GGTGGAGGAGAAGACCAG-3′ R: 5′-CGGTGGGGTTGAGGATCT-3′	58
SNAIL	F: 5′-CTTCCAGCAGCCCTACGAC-3′ R: 5′-CGGTGGGGTTGAGGATCT-3′	57
TWIST	F: 5′-AGCTACGCCTTCTCGGTCT-3′ R: 5′-CCTTCTCTGGAAACAATGACATC-3′	57.5
*TET-1*	F: 5′-TTCGTCACTGCCAACCTTAG-3′ R: 5′-ATGCCTCTTTCACTGGGTG-3′	60.5
*TET-2*	F: 5′-CACTGCATGTTTGGACTTCTG-3′ R: 5′-TGCTCATCCTCAGGTTTTCC-3′	58
*TET-3*	F: 5′-GCCCACAAGGACCAGGATAA-3′ R: 5′-CGCAGCGATTGTCTTCCTTG-3′	60

**Table 2:** Key Compounds Identified in DM Extract.

pic	RT	Area	Compound	Ref	CAS	Qual
3	27.095	2.53	5-amino-1,3-diphenyl-1H pyrazol	97544	005356-71-8	38
3	27.095	2.53	4′-Methoxyflavone	113426	004143-74-2	30
5	30.238	2.29	1-(Anilinomethylene)-2-indanone	97595	1000075-55-7	18
6	30.749	2.21	4-bromo-N-(2-chloroacetyl)pyrazole-1-carboxamide	125768	1000268-17-5	20
11	34.465	3.18	Oxazolo [3,2-E]xanthine 2,3-dihydro-2-hydroxymethyl-5,7-dimethyl-	112689	1000285-58-3	30
11	34.465	3.18	2,3-dihydro-2-hydroxymethyl-5,7-dimethyl-4H-1-Benzopyran-4-one	113438	007622-32-4	30
30	42.996	2.71	4H-1-Benzopyran-4-one, 8-methoxy-2-phenyl-	113437	026964-26-1	22
41	45.922	2.95	7-Methoxyflavone	113425	022395-22-8	30
41	45.922	2.95	2-[3-Methoxyphenyl]-4H-1-benzopyran-4-one	113433	053906-83-5	27
60	51.093	1.75	Indan-1,3-dione, 2-(1,3-dimethyl-1 H-pyrazol-4-ylmethylene)-	113316	1000316-71-1	18
62	51.635	1.70	4-[5-(4-Methoxyphenyl)-2-oxazolyl] pyridine	113289	096753-33-2	18

The “Qual” column represents the Qualitative Match Score.

## Data Availability

The data supporting the findings of this study are available within the article and its supplementary material. Any further inquiries can be directed to the corresponding author(s).

## Supplementary Materials

The following supporting information can be downloaded at: https://www.mdpi.com/article/10.3390/biom15101361/s1, Figure S1: GC-MS chromatogram of the dichloromethane–methanol (DM) extract of *Jania rubens*. Original images can be found in Supplementary File.

## Abbreviations

The following abbreviations are used in this manuscript:

CRC	Colorectal cancer
DM	Dichloromethane–methanol extract
EMT	Epithelial–mesenchymal transition
TET	Ten-Eleven Translocation enzymes
5-hmC	5-hydroxymethylcytosine
ROS	Reactive oxygen species
DHE	Dihydroethidium
PML	Promyelocytic Leukemia protein
MMP	Matrix metalloproteinase
