# Supplementary material for: Anticancer Activity of Jania rubens in HCT-116 Cells via EMT Suppression, TET Downregulation, and ROS-Mediated Cytotoxicity
Source: Biomolecules. 2025 Sep 25;15(10):1361. doi: 10.3390/biom15101361 (PMC12563589; doi:10.3390/biom15101361)

Supplementary Materials

GC-MS chromatogram of the dichloromethane–methanol (DM) extract of *Jania rubens*. Major peaks correspond to the compounds listed in Table 1, identified based on retention time (RT) and mass spectral matching with the NIST database.

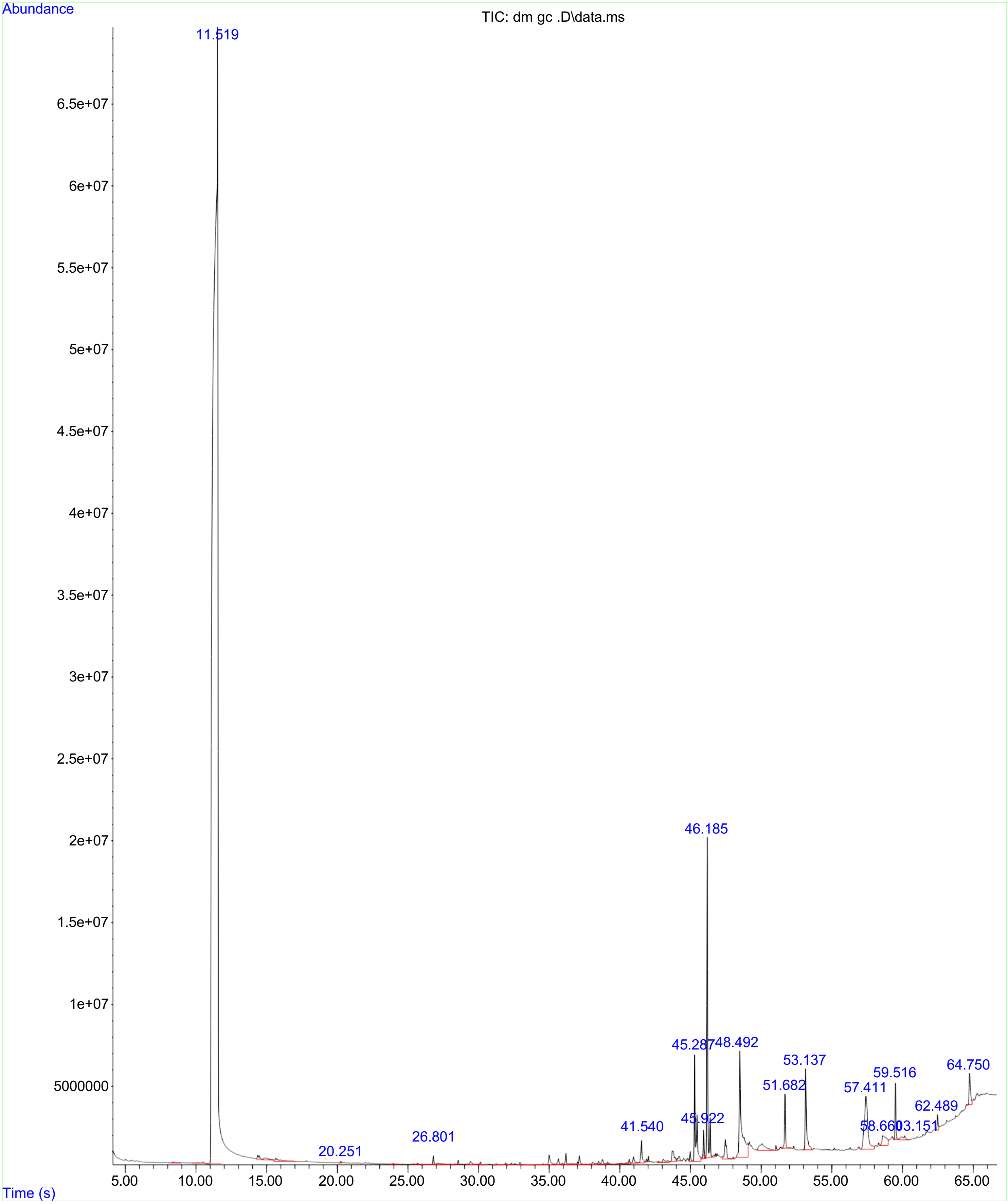

Original electrophoresis

Ladder-Ctrl- DM 250 µg/mL, DM 500 µg/mL,-1% BSA

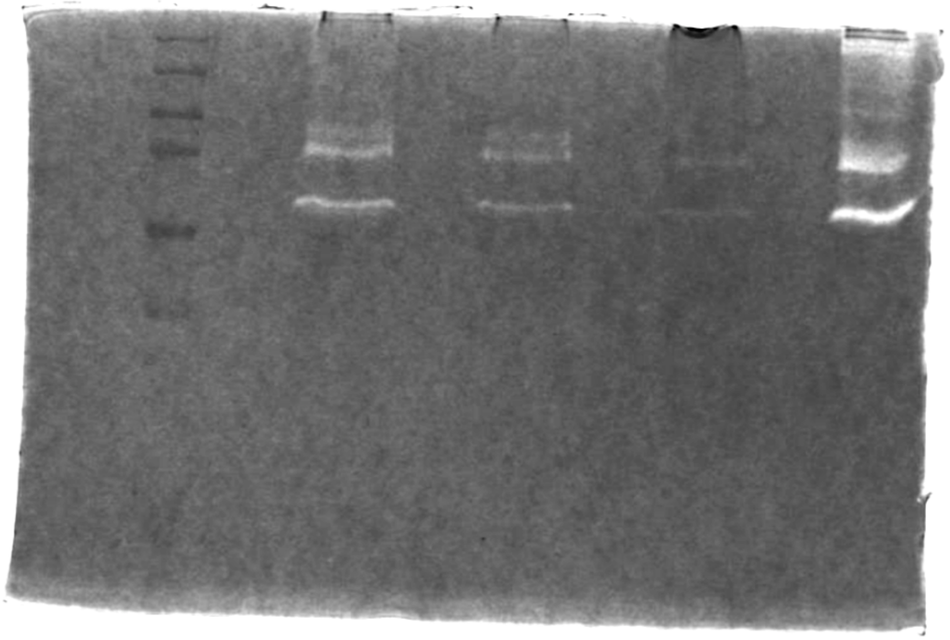

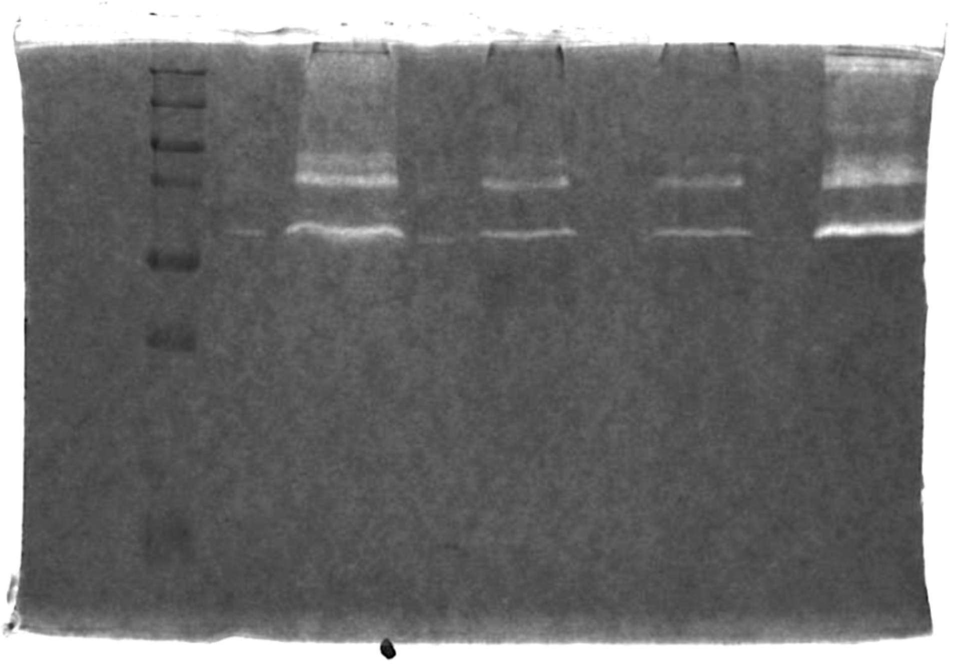

Supplement: Supplementary file 1 [file biomolecules-15-01361-s001.zip › biomolecules-3818213-Supplementary Materials.pdf]
